# Connexin channels and phospholipids: association and modulation

**DOI:** 10.1186/1741-7007-7-52

**Published:** 2009-08-17

**Authors:** Darren Locke, Andrew L Harris

**Affiliations:** 1 Department of Pharmacology and Physiology, New Jersey Medical School, University of Medicine and Dentistry of New Jersey, Newark, New Jersey 07103, USA

## Abstract

**Background:**

For membrane proteins, lipids provide a structural framework and means to modulate function. Paired connexin hemichannels form the intercellular channels that compose gap junction plaques while unpaired hemichannels have regulated functions in non-junctional plasma membrane. The importance of interactions between connexin channels and phospholipids is poorly understood.

**Results:**

Endogenous phospholipids most tightly associated with purified connexin26 or connexin32 hemichannels or with junctional plaques in cell membranes, those likely to have structural and/or modulatory effects, were identified by tandem electrospray ionization-mass spectrometry using class-specific interpretative methods. Phospholipids were characterized by headgroup class, charge, glycerol-alkyl chain linkage and by acyl chain length and saturation. The results indicate that specific endogenous phospholipids are uniquely associated with either connexin26 or connexin32 channels, and some phospholipids are associated with both. Functional effects of the major phospholipid classes on connexin channel activity were assessed by molecular permeability of hemichannels reconstituted into liposomes. Changes to phospholipid composition(s) of the liposome membrane altered the activity of connexin channels in a manner reflecting changes to the surface charge/potential of the membrane and, secondarily, to cholesterol content. Together, the data show that connexin26 and connexin32 channels have a preference for tight association with unique anionic phospholipids, and that these, independent of headgroup, have a positive effect on the activity of both connexin26 and connexin32 channels. Additionally, the data suggest that the likely in vivo phospholipid modulators of connexin channel structure-function that are connexin isoform-specific are found in the cytoplasmic leaflet. A modulatory role for phospholipids that promote negative curvature is also inferred.

**Conclusion:**

This study is the first to identify (endogenous) phospholipids that tightly associate with connexin channels. The finding that specific phospholipids are associated with different connexin isoforms suggests connexin-specific regulatory and/or structural interactions with lipid membranes. The results are interpreted in light of connexin channel function and cell biology, as informed by current knowledge of lipid-protein interactions and membrane biophysics. The intimate involvement of distinct phospholipids with different connexins contributes to channel structure and/or function, as well as plaque integrity, and to modulation of connexin channels by lipophilic agents.

## Background

Interactions of membrane proteins with membrane lipids can have dramatic functional and structural consequences [1-6]. For connexin channels this is no doubt true; lipids are likely to have modulatory influences and play fundamental roles in junctional plaque structure and/or maintenance. However, a three-dimensional channel structure of sufficient resolution to identify tightly/strongly associated membrane lipids has not yet been obtained.

The present study identifies endogenous phospholipids that remain tightly associated with purified hemichannels and with intercellular channels in junctional plaques, for channels formed by two different connexin isoforms. It also investigates the effects of specific phospholipid classes on hemichannel function. The results provide insight regarding the phospholipids that directly interact with, and affect, connexin channel activity, and identify the differences in the interactions of phospholipids with different connexin isoforms.

Gap junctions directly connect the cytoplasm of adjacent cells, enabling the sharing of ions and molecules that are essential for normal cellular development and function. Gap junction channels form by extracellular docking of two single membrane-spanning 'hemichannels' (or 'connexons'), each formed by six connexin proteins; one hemichannel is provided by each apposed cell [7]. Non-junctional unpaired hemichannels in plasma membrane also have regulated biological functions [8].

Gap junction plaques are typically large, discrete and specialized regions of plasma membrane containing lateral aggregations of intercellular channels. The lipid composition of plaques differs from surrounding plasma membrane [9-16]. However, understanding of the interaction(s) between connexin and membrane lipid remains rudimentary [15,16]. Of particular note in this context is that most connexin channel antagonists are strong lipophiles; their poor specificity for connexin over other membrane proteins, and their low efficacy, suggests their actions are membrane-mediated [17,18].

The present study was undertaken to identify endogenous phospholipids most tightly associated with channels composed of connexin26 (Cx26) or connexin32 (Cx32), with the idea that these are the most likely to have structural and/or functional roles. The strategy used was to purify stringently for the connexin channels, and then identify lipids that remained associated with them. This is therefore not a study of bulk lipid, but a study designed to reveal the identities of the lipids that connexin channels select from bulk membrane or during biogenesis with which to interact strongly and specifically. Tandem electrospray-mass spectrometry (ESI-MS/MS) was used to identify phospholipids associated with two distinct structural forms of connexin channel, that is, single hemichannels and junctional channels in isolated plaques.

Firstly, to identify phospholipids most tightly associated with hemichannels, channels with a cytoplasmic carboxyl terminal epitope tag were purified in a non-ionic detergent that maintains the hexameric form and channel function [19,20]. The channels were expressed in communication-incompetent HeLa cells using tetracycline-responsive vectors [21]. Phospholipids remaining bound to affinity-purified hemichannels after detergent solubilization and extensive washing with detergent in lipid-free high and low ionic strength buffers were considered tightly bound, and were identified by ESI-MS/MS using class-specific interpretative methods. Phospholipids specifically associated with Cx26 or Cx32 channels, or common to both isoforms, were so identified.

Secondly, to identify phospholipids essential for plaque structure, which may also include those tightly bound to the component hemichannels themselves, plaques were isolated from the same cells as above by a method that recovers highly pure gap junction plaques [22-24]. This somewhat harsh protocol, which employs two ionic detergents and one non-ionic detergent, yields highly regular arrays of junctional channels retaining the characteristic double-membrane structure. ESI-MS/MS analysis identified the lipid species unique to Cx26 plaques, Cx32 plaques, and common in both, which were distinct from those found in non-plaque membrane.

Lastly, to determine the effects of specific phospholipid classes on connexin channel activity, affinity-purified hemichannels were reconstituted into liposome membranes of defined composition, allowing study of connexin channel activity as a function of lipid environment.

Together, the data suggest an intimate involvement of different phospholipids with different connexins that contributes to modulation of channel structure, form and/or function.

## Methods

Components of the 'Tet-On' connexin expression system were from BD Biosciences (Palo Alto, CA, USA). Dulbecco's modified Eagle's medium, G418 sulfate, hygromycin, and doxycycline were from Life Technologies (Rockville, MD, USA). Agarose-conjugated anti-HA clone HA-7 mouse immunoglobulin G (IgG) was from Sigma (St Louis, MO, USA), as were all other reagents unless stated otherwise. All lipids were from Avanti Polar Lipids Inc (Alabaster, IL, USA), including cholesterol, chicken egg L-α-phosphatidic acid (PA), chicken egg L-α-phosphatidylcholine (PC), chicken egg L-α-phosphatidylethanolamine (PE), dioleoylphosphatidylethanolamine (DOPE), DOPE labeled with either nitrobenzoxadiazole or lissamine rhodamine B (DOPE-NBD and DOPE-rhodamine, respectively), chicken egg L-α-phosphatidylglycerol (PG), bovine liver L-α-phosphatidylinositol (PI) and porcine brain L-α-phosphatidylserine (PS). Detergent *n*-octyl βD glucoside (OG) (99.5% purity) was from Glycon (Luckenwalde, Germany). Bio-Gel gel filtration matrix (A-0.5 m, 100–200 mesh, exclusion limit 500 kDa) was from BioRad (Hercules, CA, USA).

### Expression of tagged connexin channels in HeLa cells

Bi-directional tetracycline-responsive expression vectors (Clontech, San Diego, CA, USA) were used to express Cx26 or Cx32 channels in HeLa cells, which have virtually no endogenous connexin expression [25]. Connexin-coding sequences were subcloned in frame with a sequence coding for a carboxyl terminal domain tag consisting of a thrombin cleavage site followed by a haemagglutinin epitope (HA, not His-Arg) and six repeats of His-Asn, that is, a HA(HN)_6 _tag. 'Tet-On' cell lines were maintained in 200 μg/ml hygromycin and 100 μg/ml G418. Cells were induced for connexin expression with 1 μg/ml doxycycline for 48 hours [21].

### Purification of tagged connexin hemichannels

Confluent 'Tet-On' HeLa cells (2,000 cm^3^) were solubilized in 50 mM NaH_2_PO_4_, 50 mM NaCl, 5 mM ethylenediaminetetraacetic acid (EDTA), 5 mM ethylene glycol tetraacetic acid (EGTA), 80 mM OG, 1 mM β-mercaptoethanol, 0.5 mM diisopropyl fluorophosphate (DFP) (Calbiochem, La Jolla, CA, USA), pH 7.5 for 2 hours at 4°C with rocking. The supernatant (100,000 *g*av, 30 minutes, 4°C) was incubated with 0.25 ml agarose-immobilized anti-HA mouse IgG overnight at 4°C with shaking. The antibody matrix was collected at 700 *g*av for 1 minute at 4°C, and washed in a fritted column with 20 ml 10 mM phosphate-buffered saline, 1 M NaCl, 80 mM OG, pH 7.4 followed by 20 ml of the same containing 138 mM NaCl. Bound material was eluted with 50 mM CH_3_COOH.Na, 0.5 M NaCl, 10 mM KCl, 1 mM EDTA, 80 mM OG, pH 4.0, and 0.6 ml fractions collected into 0.05 ml 1 M NaHCO_3_, 10 mM KCl, 80 mM OG, pH 9.0. The final pH was approx. 7.4.

### Preparation of gap junction plaques

Gap junction purification followed a procedure for isolating plaques for imaging by atomic force microscopy [22-24]. Briefly, confluent 'Tet-On' HeLa cells (4,000 cm^3^) were scraped into 8.7% w/v sucrose in 4-(2-hydroxyethyl)-1-piperazineethanesulfonic acid (HEPES)-EDTA buffer (10 mM HEPES, 2 mM EDTA, pH 7.4) containing 0.5 mM DFP, and collected by centrifugation (7,500 *g*av, 15 minutes, 4°C).

A crude membrane fraction was prepared by sonication, 3 × 10 seconds at the lowest power setting, in HEPES-EDTA buffer containing 8.7% w/v sucrose and 0.5 mM DFP. To obtain junctional membranes, discontinuous gradients were prepared in Beckman SW60Ti tubes in HEPES-EDTA buffer; the bottom step was 1 ml 50% w/v sucrose, the middle step was 2 ml 27% w/v sucrose, and sample solution was layered on top to fill. Gradients were centrifuged at 208,000 *g*av for 2 hours at 4°C, without braking. The 27/50% interface was collected, and washed twice (92,000 *g*av, 30 minutes, 4°C) with HEPES-EDTA buffer. The pellet was resuspended in 1 ml HEPES-EDTA buffer.

Pellets were solubilized using 1 vol 0.6% w/v N-lauryl sarkosine in HEPES-EDTA buffer for 30 minutes at room temperature, and then washed (92,000 *g*av, 30 minutes, 4°C) with HEPES-EDTA buffer. The centrifugation pellet was resuspended in 1 ml HEPES-EDTA buffer and solubilized in 0.6% v/v SurfactAmps Brij58 (Pierce Endogen, Rockford, IL, USA) for 30 minutes at room temperature. Membranes were collected by centrifugation (92,000 *g*av, 30 minutes, 4°C) in HEPES-EDTA buffer, and resuspended in 1 ml HEPES-EDTA buffer. To the solution was added an equal volume of 0.6% w/v saponin and, after 30 minutes incubation at room temperature, the solution was loaded into a Slide-A-Lyzer dialysis cassette (Pierce Endogen) with a molecular mass cutoff of 10 kDa. The sample was dialyzed overnight in 1 L HEPES-EDTA buffer containing 0.03% w/v saponin. Over the next 24 hours, the saponin was removed by three successive changes of dialysis buffer containing 10-fold smaller % w/v concentrations of saponin, and one further dialysis with no saponin in the dialysis solution.

After dialysis, the remaining sample was loaded on to a discontinuous gradient prepared in Beckman SW60Ti tubes in HEPES-EDTA buffer; at bottom, 1 ml 41% w/v sucrose, middle 2.5 ml 30% w/v sucrose, and sample as top step. Gradients were centrifuged at 208,000 *g*av for 2 hours at 4°C, without braking, and the 30/41% w/v interface collected, and washed twice (92,000 *g*av, 30 minutes, 4°C) with HEPES-EDTA buffer. Finally, the pellet containing the purified gap junctions was resuspended in 100 μl HEPES-EDTA buffer and used for phospholipid and/or protein analysis.

### Sample extraction and analysis by tandem electrospray-mass spectroscopy

Lipids retained in hemichannel preparations or in isolated plaques were recovered into a solution compatible for analysis by direct-infusion ESI-MS/MS. Samples were extracted with 1 vol 2:1 v/v chloroform:methanol. The chloroform layer was recovered, and saved, and the aqueous layer re-extracted with 1 vol chloroform. Chloroform layers were combined and washed with 1 vol water in triplicate. This final chloroform extract was dried and resuspended in 9:1 v/v acetonitrile:water with 10 mM NH_4_OAc. An aliquot was further diluted 1/10 for ESI-MS/MS analysis.

To detect selectively and sensitively major phospholipid classes (PA, PC, PE, PI, PS), and their class-specific fragmentation patterns, optimization of the ESI-MS/MS detection was performed using blended soy standards containing each phospholipid class. Identification of multiple molecular species within each class was possible to a limit of 10 ng/ml. Standards, and lipid(s) recovered from connexin samples, were flow-infused into an ESI-interfaced API 4000 Q-Trap mass spectrometer (Applied Biosystems, Foster City, CA, USA) at 20 μl/min. Each mass selective reaction was collected for 50 additive multichannel analyzer scans within 400 to 1,000 atomic mass units (amu). Each sample was run under positive and negative mode MS, and using class-specific MS/MS interpretative methods. Diagnostic precursor and neutral loss scans used for the different native phospholipid classes were: PA m/*z *153 amu negative precursor ion; PC m/*z *184.2 amu positive precursor ion; PE m/*z *141.0 amu positive neutral loss; PI m/*z *241.0 amu negative precursor ion; PS m/*z *88.0 amu negative neutral loss [26].

From the resulting lists of detected MS and MS/MS product ions, phospholipids were identified using manufacturer's interpretation software and interactive databases available at the LIPID Metabolites and Pathways Strategy program . In some instances, the same amu value could correspond to phospholipids with an even number of total acyl carbons (*n*) and a plasmalogen/ether acyl chain linkage, or phospholipids with *n + 1 *acyl carbons and a glycerol acyl chain linkage. The biological occurrence of acyl chains with an odd number of carbon atoms is uncertain, and not supported by a recent study of a eukaryotic lipidome with high resolution and sensitivity [27]. In view of this, these amu values were interpreted as indicating plasmalogen/ether acyl chain linkages with an even number of total carbon atoms in the acyl chains.

### Hemichannel reconstitution

Immunopurified connexin channels (approx. 1 mg/ml, with 459 mM urea and 10 mg/ml PC:PS:DOPE-fluorophore added [2:1:0.02 mol:mol]; volume 200 μl) were reconstituted into unilamellar liposomes by gel filtration using a glass HR10/30 chromatography column (bed approx. 24 ml) packed with Biogel A-0.5 m 100–200 mesh media (exclusion 500 kDa) in chilled, degassed 'urea buffer' (10 mM KCl, 10 mM Tris, 0.1 mM EDTA, 0.1 mM EGTA, 3 mM NaN_3_, pH 7.6, 459 mM urea) [19,28]. Flow rate was 9 ml/h, and connexin proteoliposomes eluted in the column void volume. The protein/lipid ratio used corresponded to an amount of connexin equivalent to less than one hemichannel per liposome so that, by design, some liposomes did not contain channels, which serve as negative controls in the Transport Specific Fractionation (TSF) activity assay.

### Transport Specific Fractionation assay

TSF was used to assess the molecular activity of reconstituted connexin hemichannels [19,20,29-37]. TSF fractionates liposomes into two populations within an iso-osmolar density gradient, based on channel permeability to urea and sucrose, uncharged solutes that have different density at iso-osmolar concentrations (urea buffer density (ρ) 1.0055 g/ml; when 400 mM sucrose replaces urea, ρ 1.0511 g/ml: both 500 mOsm/kg). Liposomes are formed in, and entrap, urea-containing solution, and are then centrifuged (300,000 *g*av, 3 hours, 37°C, slow acceleration, no brake) through linear iso-osmotic TSF density gradients formed from urea and sucrose buffers. Equilibration of these solutes across the liposome through an open hemichannel occurs rapidly and increases the density of the liposome. TSF, therefore, reports 'all-or-nothing' permeation of urea and sucrose through connexin channels on a per-liposome basis. Fractionation of liposomes into bands is monitored by the fluorescence of DOPE- NBD (λ_ex _460 nm, λ_em _534 nm) or DOPE- rhodamine (λ_ex _570 nm, λ_em _590 nm) in the liposome membrane. The typical positions of the upper and lower bands are centered at ρ 1.02 g/ml and ρ 1.04 g/ml, respectively. Liposomes that contain channels permeable to one solute but not the other undergo osmotically driven shrinkage that results in a band of intermediate density, ρ 1.03 g/ml. Bands were assigned as upper, intermediate, and lower on the basis of density, as determined by refractometry at 25°C [31]. In the present study, intermediate bands were not observed (not shown).

## Results

Homomeric Cx26 or homomeric Cx32 channels were expressed in communication-incompetent HeLa cell lines using tetracycline-responsive vectors [21]. Hemichannels were purified by immunoaffinity chromatography using the carboxyl terminal epitope tag in a non-ionic detergent, OG, which maintains the hexameric structure of the hemichannel and its channel function [19,20]. The purification protocol involved no additional lipid and extensive rinsing of immobilized connexin with OG.

Gap junction plaques were purified using a protocol based on the original method of Fallon and Goodenough [38], taking advantage of the detergent (sarkosyl and Brij) insolubility of condensed gap junction plaques. Adaptations include saponin treatment, which preferentially solubilizes cholesterol, and subsequent slow dialysis against decreasing saponin concentrations, which improve structural clarity of the junctional plaques [22-24]; such treatments substantially delipidate the junctional plaques.

### Phospholipids associated with hemichannels and gap junction plaques

Using class-specific interpretative methods, ESI-MS/MS was used to identify phospholipids remaining bound to hemichannels after affinity purification. These phospholipids were categorized as unique or common to homomeric Cx26 or homomeric Cx32 hemichannels. Phospholipids in isolated gap junction plaques composed of Cx26 or Cx32 junctional channels were similarly identified and categorized.

Phospholipids recovered as potential artifacts of isolation were identified and excluded; the hemichannel and plaque isolation procedures were applied to the same cell lines not induced to express connexin. Lipid species found in these negative control samples (for example, non-specifically binding to the immunoaffinity beads) were excluded from the following results. However, since some of these lipids might also interact strongly with connexin channels, and only reported positive findings have meaning, absence of specific phospholipid species in the data do not mean there is no interaction with connexin.

The purified hemichannels and isolated plaques were characterized by SDS-PAGE and Western blotting, gold stain of protein blots, and in-gel zinc exclusion stain to confirm purity and confirm connexin expression only after doxycycline induction [21]. Cx45, sometimes expressed endogenously at low levels by HeLa cells [25], was not found (not shown).

The ESI-MS/MS analyses were restricted to five major phospholipid groups: PA, PC, PE, PI and PS.

**Figure 1:**
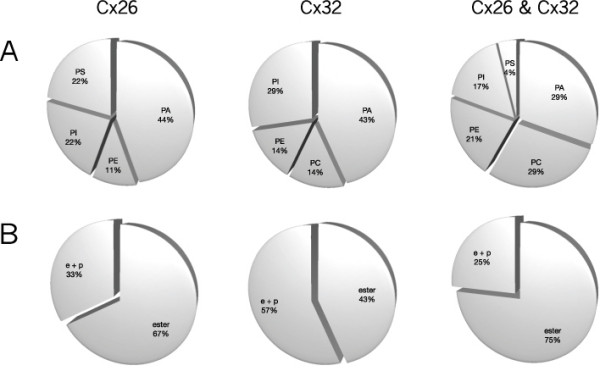
**Phospholipid headgroups and glycerol-alkyl chain linkage for hemichannels**. Distribution by class and source, **(a) **headgroups and **(b) **glycerol-alkyl chain linkage, identified as unique to Cx26 (left), unique to Cx32 (middle), or common to both Cx26 and Cx32 (right). PA = phosphatidic acid; PC = phosphatidylcholine; PE = phosphatidylethanolamine; PI = phosphatidylinositol; PS = phosphatidylserine; *e+p *= alkyl ether (*e*) and plasmenyl (*p*) glycerol-alkyl chain linkage; ester = ester (conventional) glycerol-alkyl chain linkage. The data on headgroup identity presented here (and in Figure 2) are included in Figures 3 and 4, with the chain length and desaturations of each lipid form also shown.

**Figure 2:**
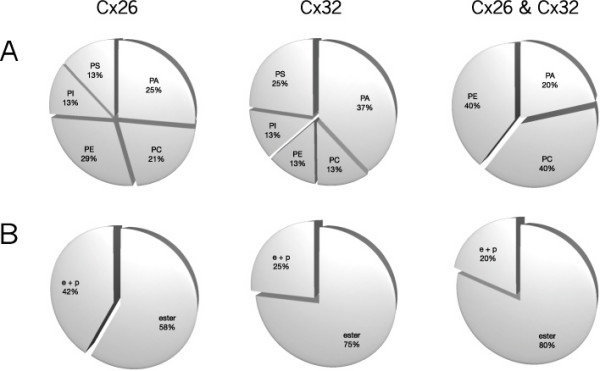
**Phospholipid headgroups and glycerol-alkyl chain linkage for junctional plaques**. Distribution by class and source, **(a) **headgroups and **(b) **glycerol-alkyl chain linkage, identified as unique to Cx26 (left), unique to Cx32 (middle), or common to both Cx26 and Cx32 (right). Abbreviations as in Figure 1. The data on headgroup identity presented here (and in Figure 1) are included in Figures 3 and 4, with the chain length and desaturations of each lipid form also shown.

**Figure 3:**
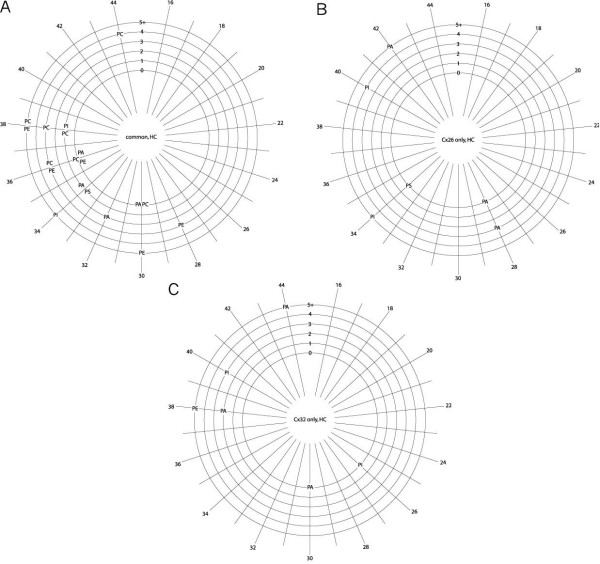
**Phospholipids associated with purified hemichannels identified by class, chain length and number of desaturations**. Radial distribution showing total number of carbon atoms in the fatty acid acyl chains of phospholipids common to both Cx26 and Cx32 **(a)**, unique to Cx26 **(b) **or unique to Cx32 **(c)**. The numbers of fatty acid acyl chain desaturations (zero to five or more) are shown on the radial arms. Abbreviations as in Figure 1. The species shared between **(a) **and **(c) **differ in glycerol-alkyl chain linkage.

**Figure 4:**
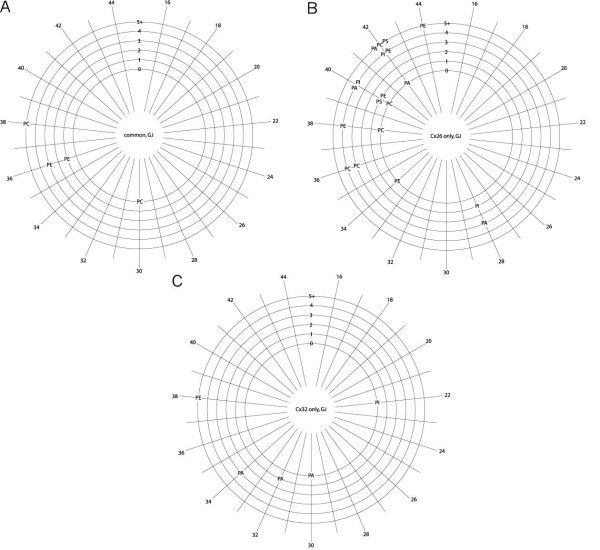
**Phospholipids associated with isolated plaques identified by class, chain length and number of desaturations**. Radial distribution showing total number of carbon atoms in the fatty acid acyl chains of phospholipids common to both Cx26 and Cx32 **(a)**, unique to Cx26 **(b) **or unique to Cx32 **(c)**. The numbers of fatty acid chain desaturations (zero to five or more) are shown on the radial arms. Abbreviations as in Figure 1.

**Figure 5:**
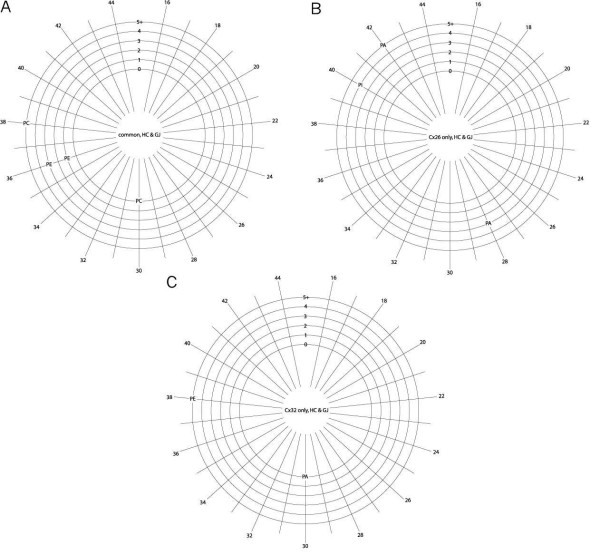
**Phospholipids associated with both hemichannels and plaques identified by class, chain length and number of desaturations**. Radial distribution showing total number of carbon atoms in the fatty acid acyl chains of phospholipids common to both Cx26 and Cx32 **(a)**, unique to Cx26 **(b) **or unique to Cx32 **(c)**. The numbers of fatty acid chain desaturations (zero to five or more) are shown on the radial arms. Abbreviations as in Figure 1.

Each phospholipid was identified by headgroup, glycerol-alkyl chain linkage, total number of carbon atoms in the acyl chains, and degree of acyl chain desaturation. The phospholipids (Tables 1, 2, 3, 4 and 5) are categorized as found in (a) hemichannels or (b) plaques, and (for a and b) as being (i) unique to Cx26, (ii) unique to Cx32 or (iii) common to Cx26 and Cx32 samples. To be identified as phospholipids in 'common' to both Cx26 and Cx32, there had to be an exact match for all the analyzed parameters (headgroup, linkage, acyl chain carbon atom content, and desaturation). The data from Tables 1 to 5 are represented graphically in Figures 1, 2, 3, 4 and 5, with the phospholipids in each category organized by headgroup and glycerol-alkyl chain linkage (Figure 1 for hemichannels, Figure 2 for plaques), and by headgroup, total acyl chain carbon content, and number of desaturations (Figure 3 for hemichannels, Figure 4 for plaques, Figure 5 for hemichannel and plaques in common). Note, in this report, the term 'acyl chain length' refers to the total number of carbon atoms in *both *acyl chains (*sn*-1 and *sn*-2), not the lengths of the individual acyl chains.

**Table 1:** Tandem electrospray-mass spectroscopy identification of phospholipids associated with connexin channels – PA.

amu observed	amu expected	PA
(a) i – Cx26 unique, hemichannels		
407.30	407.22	16:1 lyso
575.50	575.40	28:0*p*
585.30ϕ	585.35	28:3
769.50ϕ	769.48	42:9
		
(b) i – Cx26 unique, plaques		
431.30	431.22	18:3 lyso
481.20	481.23	22:6 lyso
585.30ϕ	585.35	28:3
751.50	751.52	40:4
769.50ϕ	769.48	42:9
787.68	787.62	42:0
		
(a) ii – Cx32 unique, hemichannels		
603.40ϕ	603.43	30:1*e *or 30:0*p*
711.50	711.53	38:3*e *or 38:2*p*
801.60	801.54	44:7
		
(b) ii – Cx32 unique, plaques		
603.40ϕ	603.43	30:1*e *or 30:0*p*
645.40	645.44	32:1
669.40	669.44	34:3
		
(a) iii – common to Cx26 and Cx32, hemichannels		
449.40	449.30	20:0 lyso
463.30	463.28	20:1 lyso
547.40	547.37	26:0p lyso
605.50	605.45	30:0*e*
643.50	643.43	32:2
673.40	673.48	34:1
687.60	687.53	36:1*e *or 36:0*p*
		
(b) iii – common to Cx26 and Cx32, plaques		
423.30	423.28	18:0*e *lyso

PA phospholipids in (a) hemichannel-detergent micelles and in (b) junctional plaques containing (i) only Cx26, (ii) only Cx32, or (iii) that are in both Cx26 and Cx32 samples, after removal of phospholipids found in negative control samples that did not contain either connexin. Tables are collated by unique mass (amu), with tolerance ± 0.10 amu from expected. Species highlighted with ϕ are found in both isolated plaques and hemichannel preparations containing the same connexin isoform. PA = phosphatidic acid; *e *= alkyl ether glycerol-alkyl chain linkage; *p *= plasmenyl glycerol-alkyl chain linkage; lyso = lysophospholipid. Absence of *e *or *p *denotation indicates conventional ester glycerol-alkyl chain linkage. *n *equals two to seven tandem electrospray-mass spectroscopy runs per sample.

**Table 2:** Tandem electrospray-mass spectroscopy identification of phospholipids associated with connexin channels – PC.

amu observed	amu expected	PC
(a) i – Cx26 unique, hemichannels		
		None
		
(b) i – Cx26 unique, plaques		
480.32	480.34	16:0*e *lyso
766.64	766.57	36:5*e *or 36:4*p*
768.64	768.59	36:4*e *or 36:3*p*
802.72	802.66	38:1*e *or 38:0*p*
846.64	846.69 846.63	40:0 42:6*p*
		
(a) ii – Cx32 unique, hemichannels		
520.30	520.34	18:2 lyso
		
(b) ii – Cx32 unique, plaques		
544.24	544.34	20:4 lyso
		
(a) iii – common to Cx26 and Cx32, hemichannels		
706.50ϕ	706.53	30:0
784.50	784.58	36:3
790.70	790.63	36:0
808.50ϕ	808.58	38:5
812.60	812.61	38:3
816.60	816.64	38:1
894.70	894.69	44:4
		
(b) iii – common to Cx26 and Cx32, plaques		
706.50ϕ	706.53	30:0
808.50ϕ	808.58	38:5

PC phospholipids in (a) hemichannel-detergent micelles and in (b) junctional plaques containing (i) only Cx26, (ii) only Cx32, or (iii) that are in both Cx26 and Cx32 samples, after removal of phospholipids found in negative control samples that did not contain either connexin. Tables are collated by unique mass (amu), with tolerance ± 0.10 amu from expected. Species highlighted with ϕ are found in both isolated plaques and hemichannel preparations containing the same connexin isoform. PC = phosphatidylcholine; *e *= alkyl ether glycerol-alkyl chain linkage; *p *= plasmenyl glycerol-alkyl chain linkage; lyso = lysophospholipid. Absence of *e *or *p *denotation indicates conventional ester glycerol-alkyl chain linkage. *n *equals two to seven tandem electrospray-mass spectroscopy runs per sample.

**Table 3:** Tandem electrospray-mass spectroscopy identification of phospholipids associated with connexin channels – PE.

amu observed	amu expected	PE
(a) i – Cx26 unique, hemichannels		
510.30	510.35	20:0 lyso
		
(b) i – Cx26 unique, plaques		
480.32	480.30	18:1 lyso
502.32	502.29	20:4 lyso
706.64	706.57	34:0*e*
768.64	768.55	38:4
802.72	802.63	40:1
808.72	808.62	42:4*p*
846.64	846.60	44:7
		
(a) ii – Cx32 unique, hemichannels		
766.60ϕ	766.53	38:5
		
(b) ii – Cx32 unique, plaques		
766.60ϕ	766.53	38:5
		
(a) iii – common to Cx26 and Cx32, hemichannels		
630.50	630.41	28:3
654.40	654.41	30:5
742.50ϕ	742.53	36:3
746.50ϕ	746.57	36:1
760.50	760.49	38:8
		
(b) iii – common to Cx26 and Cx32, plaques		
742.50ϕ	742.53	36:3
746.50ϕ	746.57	36:1

PE phospholipids in (a) hemichannel-detergent micelles and in (b) junctional plaques containing (i) only Cx26, (ii) only Cx32, or (iii) that are in both Cx26 and Cx32 samples, after removal of phospholipids found in negative control samples that did not contain either connexin. Tables are collated by unique mass (amu), with tolerance ± 0.10 amu from expected. Species highlighted with ϕ are found in both isolated plaques and hemichannel preparations containing the same connexin isoform. PE = phosphatidylethanolamine. *e *= alkyl ether glycerol-alkyl chain linkage; *p *= plasmenyl glycerol-alkyl chain linkage; lyso = lysophospholipid. Absence of *e *or *p *denotation indicates conventional ester glycerol-alkyl chain linkage. *n *equals two to seven tandem electrospray-mass spectroscopy runs per sample.

**Table 4:** Tandem electrospray-mass spectroscopy identification of phospholipids associated with connexin channels – PI.

amu observed	amu expected	PI
(a) i – Cx26 unique, hemichannels		
825.50	825.45	34:6
897.68ϕ	897.58	40:5*e *or 40:4*p*
		
(b) i – Cx26 unique, plaques		
751.50	751.43	28:1
897.68ϕ	897.58	40:5*e *or 40:4*p*
925.68	925.61	42:4*e*
		
(a) ii – Cx32 unique, hemichannels		
711.50	711.44	26:0*e*
899.60	899.60	40:4*e *or 40:3*p*
		
(b) ii – Cx32 unique, plaques		
669.40	669.36	22:0
		
(a) iii – common to Cx26 and Cx32, hemichannels		
583.40	583.32	18:0*p *lyso
585.30	585.34	18:0*e *lyso
825.50	825.45	34:6
875.70	875.61	38:2*e *or 38:1*p*
		
(b) iii – common to Cx26 and Cx32, plaques		
		None

PI phospholipids in (a) hemichannel-detergent micelles and in (b) junctional plaques containing (i) only Cx26, (ii) only Cx32, or (iii) that are in both Cx26 and Cx32 samples, after removal of phospholipids found in negative control samples that did not contain either connexin. Tables are collated by unique mass (amu), with tolerance ± 0.10 amu from expected. Species highlighted with ϕ are found in both isolated plaques and hemichannel preparations containing the same connexin isoform. PI = phosphatidylinositol; *e *= alkyl ether glycerol-alkyl chain linkage; *p *= plasmenyl glycerol-alkyl chain linkage; lyso = lysophospholipid. Absence of *e *or *p *denotation indicates conventional ester glycerol-alkyl chain linkage. *n *equals two to seven tandem electrospray-mass spectroscopy runs per sample.

**Table 5:** Tandem electrospray-mass spectroscopy identification of phospholipids associated with connexin channels – PS.

amu observed	amu expected	PS
(a) i – Cx26 unique, hemichannels		
510.30	510.31	18:0*p *lyso
762.60	762.52	34:0
		
(b) i – Cx26 unique, plaques		
480.32	480.27	16:0*p *lyso
580.32	580.36	22:0 lyso
846.64	846.62 846.56	40:1 42:6*e*
		
(a) ii – Cx32 unique, hemichannels		
		None
		
(b) ii – Cx32 unique, plaques		
482.32	482.28	16:0*e *lyso
544.24	544.26	20:4 lyso
		
(a) iii – common to Cx26 and Cx32, hemichannels		
760.50	760.51	34:1
		
(b) iii – common to Cx26 and Cx32, plaques		
		None

PS phospholipids in (a) hemichannel-detergent micelles and in (b) junctional plaques containing (i) only Cx26, (ii) only Cx32, or (iii) that are in both Cx26 and Cx32 samples, after removal of phospholipids found in negative control samples that did not contain either connexin. Tables are collated by unique mass (amu), with tolerance ± 0.10 amu from expected. Species highlighted with ϕ are found in both isolated plaques and hemichannel preparations containing the same connexin isoform. PS = phosphatidylserine; *e *= alkyl ether glycerol-alkyl chain linkage; *p *= plasmenyl glycerol-alkyl chain linkage; lyso = lysophospholipid. Absence of *e *or *p *denotation indicates conventional ester glycerol-alkyl chain linkage. *n *equals two to seven tandem electrospray-mass spectroscopy runs per sample.

Inspection of the data showed evidence of some hydrolysis in the form of unnaturally occurring lysolipids, for example, with saturated acyl chains. These lipids are included in the tables, and represented in Figures 1 and 2, as the headgroup and linkage data are valid, but are omitted from Figures 3 to 5, as the information about the acyl chains (carbon number and saturation) is potentially artifactual.

#### Headgroup and glycerol-alkyl chain linkage: hemichannels

Some species of each of the phospholipid classes analyzed were common to Cx26 and Cx32 hemichannels (Figure 1a, right). Phospholipids specific for Cx26 hemichannels excluded PC (Figure 1a, left) and phospholipids specific for Cx32 hemichannels excluded PS (Figure 1a, middle). Thus, no unique species of PS were associated with Cx32 hemichannels, and no unique species of PC were associated with Cx26 hemichannels.

The proportion of anionic phospholipid species found uniquely associated with Cx26 hemichannels or with Cx32 hemichannels was much greater than that associated with both types of hemichannels, in which approx. 50% of the number of phospholipid species identified were zwitterionic. The proportion of unique ether-linked phospholipid species (those with alkyl ether (*e*) or vinyl ether/plasmenyl (*p*) glycerol-alkyl chain linkage; noted in tables and figures as '*e*', '*p*' or '*e*+*p*') was substantially greater in the Cx32 hemichannel preparations than in the Cx26 hemichannel preparations (Figure 1b).

#### Headgroup and glycerol-alkyl chain linkage: plaques

A small number of zwitterionic phospholipids were common to both Cx26 and Cx32 plaques, consisting of species of PC, PE and PA (Figure 2a, right). Although phospholipids unique to Cx26 and Cx32 plaques included members of all five phospholipid classes, these were in different proportions, with Cx32 plaques favoring anionic lipids (Figure 2a, left and center). Thus, all the identified species of PI and PS in Cx26 or Cx32 plaques were unique to each connexin isoform.

In contrast to the phospholipids common to Cx26 and Cx32 hemichannel preparations (Figure 1a, right), the phospholipids common to Cx26 and Cx32 plaques excluded PI and PS. The proportion of ether-linked phospholipids unique to Cx26 plaques was greater than that unique to Cx32 plaques (Figure 2b). The differences in the phospholipid species found uniquely in Cx26 and Cx32 plaques suggest that the integrity of plaque structures formed by these two connexins have distinct phospholipid requirements.

#### Headgroup, acyl chain length and saturation: hemichannels

Graphical representation of the phospholipid species associated with hemichannels, delineated by headgroup, acyl chain length (total number of acyl chain carbons), and number of desaturations in the fatty acid acyl chains, reveals greater detail (Figure 3). A large number of phospholipid species were common to both Cx26 and Cx32 hemichannel preparations (Figure 3a), these having 28 to 38 acyl carbons (with a single exception of 44 carbons; median 36) (Table 6). There was a broad range of saturations, which did not correlate with chain length.

**Table 6:** Differences in phospholipids associated with connexin hemichannels.

Hemichannel	Headgroup	Linkage	Carbon atoms (number)	Saturation
Cx26	PE, PA, PI, PS. No unique species of PC. Predominantly anionic.	Fewer ether-linkages than unique to Cx32.	Range 28 to 42. Mean 34. Median 34.	Bimodal distribution.
Cx32	PC, PE, PA, PI. No unique species of PS. Mostly anionic, less than for Cx26.	More ether-linkages than in common or unique to Cx26.	Range 26 to 44. Mean 36. Median 38.	Broad distribution. Shorter chains more saturated.
Cx26 and Cx32	PC, PE, PA, PI, PS. Zwitterionic species approx. 50%, more than are unique to Cx26 or Cx32.	Similar ether-linkages to those unique to Cx26.	Range 28 to 38 + 44. Mean 35. Median 36.	Broad distribution.

Abbreviations as previously.

The range of acyl carbons was similar for lipids unique to Cx26 and to Cx32 hemichannels, ranging from 28 to 42 (median 34) for the former, and from 26 to 44 (median 38) for the latter. The phospholipids unique to Cx26 hemichannels tended to be either fully saturated or highly desaturated (Figure 3b). The amount of desaturation in phospholipids unique to Cx32 hemichannels (Figure 3c) was more evenly distributed, and phospholipids with longer chain lengths were more desaturated.

#### Headgroup, acyl chain length and saturation: plaques

The corresponding graphical representations of the specific phospholipid species associated with plaques are shown in Figure 4. There were many fewer phospholipid species common to Cx26 and Cx32 plaques (Figure 4a) than were in common in the corresponding hemichannel preparations (Figure 3a). The total carbon content of the acyl chains ranged from 30 to 38 (median 36), with the longer chains being more desaturated (Table 7).

**Table 7:** Differences in phospholipids associated with connexin junctional plaques.

Plaque	Headgroup	Linkage	Carbon atoms (number)	Saturation
Cx26	PC, PE, PA, PI, PS. Approx. 50% anionic.	More ether-linkages than unique to Cx32.	Range 28 to 44. Mean 39. Median 40.	Broad distribution.
Cx32	PC, PE, PA, PI, PS. More anionic than Cx26.	Fewer ether-linkages than unique to Cx26.	Range 22 to 38. Mean 31. Median 32.	Broad distribution, shorter chains more saturated.
Cx26 and Cx32	PC, PE, PA. No unique species of PI and PS. Predominantly zwitterionic.	Fewer ether-linkages than unique to Cx26, similar to Cx32.	Range 30 to 38. Mean 35. Median 36.	Broad distribution, shorter chains more saturated.

Abbreviations as previously.

A relatively large number of phospholipid species were specific for Cx26 plaques. Their chain lengths were skewed to longer lengths (range 28 to 44; median 40) (Figure 4b), compared with those for the Cx32 plaques (range 22 to 38; median 32) (Figure 4c) or those common to plaques of both connexins. However, phospholipids unique to Cx26 plaques tended to be more desaturated than those in Cx32 plaques. For the lipids unique to Cx32 plaques, the degree of desaturation correlated with chain length.

#### Headgroup, acyl chain length and saturation: common to hemichannels and plaques

Tables 6 and 7, respectively, present a summary of the phospholipid differences between Cx26 and Cx32 *and *their hemichannel versus plaque structural forms. For each connexin isoform, a small number of phospholipid species was found in both hemichannel and plaque preparations (Figure 5; designated by ϕ in Tables 1 to 5). The phospholipids in common in hemichannel preparations and isolated junctional plaques for both connexins were two highly saturated species of relatively short acyl chain length PC, one median acyl chain length polyunsaturated species of PC, and two species of moderately saturated median acyl chain length PE (Figure 5a). Notably, these are all zwitterionic. Unique to Cx26 in both structural forms were two species of polyunsaturated PA, one of short and one of long acyl chain length, and one long acyl chain length polyunsaturated species of PI (Figure 5b). Unique to Cx32 in both structural forms were a species of highly saturated relatively short acyl chain length PA, and a polyunsaturated species of medium acyl chain length PE (Figure 5c). Thus, the anionic phospholipid species common to both the hemichannels and plaques appear to be connexin-specific.

### Phospholipid composition modulates hemichannel activity

To investigate the effect of lipid environment on channel function, Cx26 and Cx32 hemichannels were reconstituted into liposomes of defined phospholipid composition. Hemichannels are more accessible for functional study than junctional channels. The functional properties of junctional channels are typically predictable from those of the component hemichannels [39-43].

Channel activity was assessed by a well-established liposome fractionation technique [19,20,29-37]. This activity assay separates liposomes in iso-osmolar gradients based on differences in liposome density caused by channel permeability to large molecules (here, to urea and sucrose). These uncharged solutes non-selectively permeate open connexin channels and have different density at iso-osmolar concentrations.

**Figure 6:**
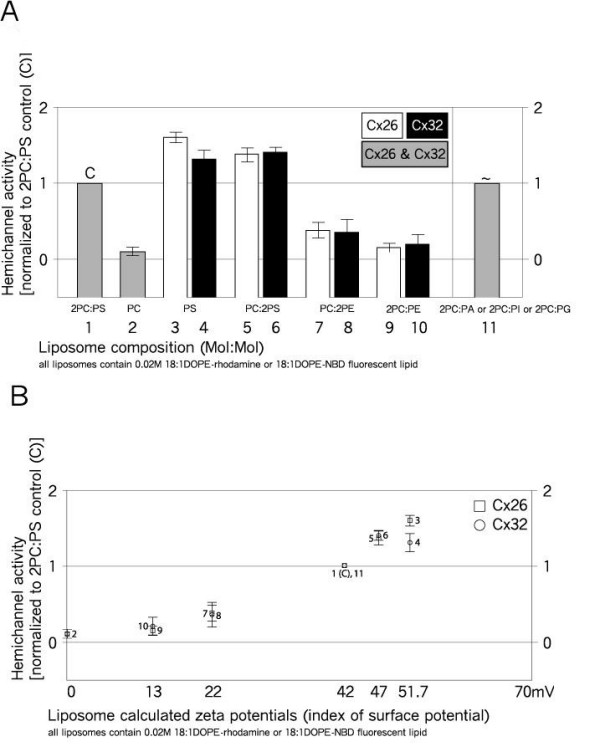
**Effect of different phospholipids on connexin hemichannel activity in liposomes**. The activity of homomeric Cx26 (squares) and Cx32 (circles) channels was assessed in isoosmotic density centrifugation gradients to separate liposomes containing functional channels from those that do not on the basis of channel permeability to gradient osmolytes (Transport Specific Fractionation, TSF). Activity is measured as the percentage of liposomes containing functional channels compared with the control **(a)**. Control liposome composition was (mol:mol) 2PC:PS and approx. 0.02 mol% DOPE-NBD or DOPE-rhodamine. EPC = ethylphosphatidylcholine; DOPE = dioleoylphosphatidylethanolamine; DOPE-NBD and DOPE-rhodamine = DOPE headgroup labeled with nitrobenzoxadiazole (NBD) or lissamine rhodamine B (rhodamine), respectively. Other abbreviations as in Figure 1. **(b) **The activity data from **(a) **was plotted as a function of calculated zeta potential. Zeta potentials were calculated from McLaughlin [47] and Winiski *et al*. [48], adjusted for TSF conditions of pH 7.6 and 46.4 mM salt. pK of 8.5 was used to calculate the charge of PE (see text). For **(b)**, numbers 1 to 11 next to the data points refer to the compositions/channels shown in **(a)**. *n *equals three to six trials per data point.

The reference liposome membrane composition for these studies was 2PC:PS (mol:mol), with approx. 0.02 mol% DOPE-rhodamine or DOPE-NBD added for visualization of liposomes (Figure 6a, bar 1). Elimination of the anionic charge of the liposome membranes contributed by PS resulted in near elimination of urea-sucrose permeability of both Cx26 and Cx32 channels (Figure 6a, bar 2). On the other hand, increasing the anionic charge of the membrane by elimination of the PC (that is, pure PS membranes) significantly increased activity of both types of channels, with the effect on Cx26 channels somewhat greater (Figure 6a, bars 3 and 4). These findings formed the basis of an investigation of the role of the phospholipid headgroup charge, and its basis, for connexin channel function.

A predominance of PS over PC (PC:2PS) only slightly reduced the activity from that in pure PS membranes (Figure 6a, bars 5 and 6). Replacing PS with PE (PC:2PE), which is almost completely deprotonated at the pH of this study (pH 7.6) and thus carrying only weak anionic charge, substantially reduced channel activity relative to the reference (Figure 6a, bars 7 and 8 versus bar 1), but slightly increased activity relative to pure PC membranes (Figure 6a, bar 2). This increase may be due to the weak anionic charge of PE (estimates of the pK of the amine of PE range from 8.5 to 9.6 [44-46], corresponding to 11% and 1% negative charge at pH 7.6, respectively). These results are also consistent with anionic membrane charge positively influencing connexin hemichannel activity. Additional PC (2PC:PE) further reduced the activity (Figure 6a, bars 9 and 10), supporting this idea.

To investigate whether the origin of the anionic charge affected activity, 2PC:P*X *mixtures of PC and either PA, PI or PG were tested. All gave similar results, essentially identical to the activity of the reference (Figure 6a, bar 11 versus bar 1). This result suggests that the observed differences in TSF channel activity are not specific to the phospholipid headgroup carrying the anionic charge, but that it is the charge itself that is important.

To characterize the relationship between membrane surface charge and connexin channel activity, the zeta potential for each of the liposome membrane compositions in Figure 6a was calculated according to McLaughlin [47] and Winiski *et al*. [48]. The zeta potential is the average electrostatic potential at the hydrodynamic plane of shear of a membrane, which is 2 Å in 100 mM salt. Hemichannel activity for homomeric Cx26 and Cx32 channels increased monotonically with the magnitude of the calculated negative zeta potential of the liposome membrane (Figure 6b). Measurement of channel activity in this study is unaffected by the effects of surface charge on local permeant ion concentration, since the flux being measured is to uncharged solutes.

**Figure 7:**
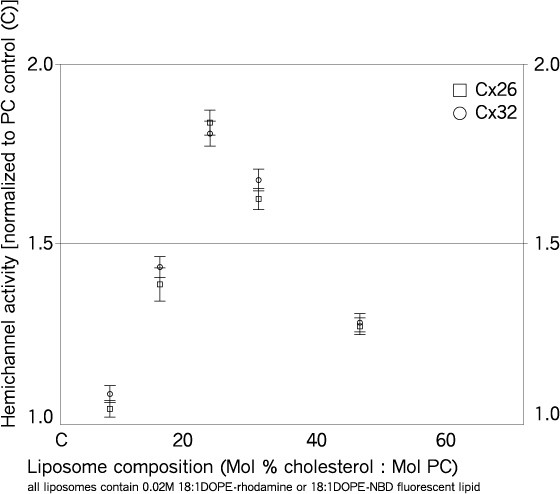
**Connexin channel activity as a function of mol% cholesterol content**. Channel activity of homomeric Cx26 (squares) and Cx32 (circles) channels was assessed by transport-specific fractionation (as in Figure 6). Channel activity was normalized to activity in zero cholesterol (PC:0.02DOPE-fluorophore, mol:mol). Data points are at 8, 16, 23, 31 and 47 mol% cholesterol. CHOL = cholesterol; PC = phosphatidylcholine. *n *equals three to six trials per data point.

Cholesterol is a major component of eukaryotic plasma membrane and is enriched in most gap junctions [9-16]. Effects of cholesterol on the activity of connexin channels in zwitterionic phospholipid (PC) were determined. The mol% cholesterol:PC was varied over a wide range and channel activity determined by TSF. The data show that channel activity was biphasic with increasing cholesterol:PC ratio, with maximum activity at approx. 25 mol% cholesterol (Figure 7). The effects of cholesterol on Cx26 and Cx32 channel activity were essentially identical.

## Discussion

Like all membrane proteins, connexin channels are subject to thermodynamic, chemical and physical influences of the lipid bilayer in which they are embedded. Unlike most membrane proteins, they exist either within junctional plaques or as unapposed hemichannels in plasma membrane. The plaques are structurally and compositionally distinct from the surrounding plasma membrane [9-15]. Unapposed hemichannels in plasma membrane have been reported as being present in distinct lipid microdomains, perhaps being trafficked to plaques [49-51], whose lipid composition may be, to some extent, specific for different connexin isoforms [50]. Additionally, MS analysis of Cx26 and Cx32 [33] has identified post-translational modifications likely to impart significant interactions of one or more of the cytoplasmic domains of these connexin isoforms with membranes, including γ-carboxylation of glutamic acid residues in the loop (coordinates with Ca^2+ ^enabling domain-level membrane interaction [52]), and palmitoylation of the tail of Cx32 but not Cx26 (implicated in 'raft' microdomain sorting [53]).

Endogenous lipids retained by the hemichannels or plaques throughout extensive detergent extraction are here regarded as tightly bound. Lipids tightly associated with membrane proteins typically have modulatory and structural effects (refer to [1-3]). A prominent example is the KcsA channel, which, when purified in *n*-dodecyl βD maltoside (of the same family as OG), retains anionic lipids, particularly PG, which are essential for channel function [54-56].

To identify specific phospholipids tightly associated with Cx26 and Cx32 channels, the present study obtained highly pure functional connexin hemichannels stabilized in non-ionic detergent. During isolation, hemichannels were extensively washed with high and low ionic strength buffers containing detergent at approx. three to five times its critical micelle concentration (CMC) and no lipid. The rationale is that phospholipids remaining associated with connexin hemichannels throughout these conditions are those that interact strongly with the protein and, therefore, are likely to have direct and important structural and/or functional interactions. Applying this strategy to hemichannels formed by two different connexins allowed identification of phospholipids that associate uniquely and in common with each.

A second goal was to identify the specific phospholipids involved in maintenance and/or the integrity of the junctional plaque, and to determine whether these species differ for plaques composed of the two different connexin isoforms. Fortunately, extensive efforts by others to obtain highly pure junctional plaques for high resolution structural studies of the intercellular channels have identified protocols that highly enrich plaque membrane and remove substantial lipid from junctional plaques, without disrupting overall plaque structure. Analogous studies of lipids associated with isolated purple membrane have yielded information about the lipids that are involved in bacteriorhodopsin structure and function [57,58].

In this study, the connexin protein itself is the determinant of what lipids are found in the samples. For this reason, the lipids may be of quite low abundance relative to the bulk composition of the plasma membrane. In a sense, the protein selects by its particular affinities among the components of the membrane which ones with which to tightly interact.

For the plaque preparations, there is, in theory, a possibility of lipid exchange between the plaques and contaminating non-plaque membrane at the point of solubilization by detergent. Detergent (Brij and sarkosyl) is added after junctional membranes are separated from non-junctional membranes using discontinuous sucrose centrifugation gradients. With the addition of detergent, contaminant lipid exchange, if it occurred, would be between detergent micelles containing lipid solubilized from residual non-junctional membrane and the plaques, which undergo substantial delipidation and condensation in this process. Even if this occurred, phospholipid species found in negative control samples were excluded from the results to eliminate non-specific (that is, non-connexin) lipid interactions.

A question arises as to the whether the sources of the connexin in the hemichannel preparations and the isolated plaques are distinct or overlap. The former may contain hemichannels from intracellular compartments in addition to unapposed hemichannels in plasma membrane and hemichannels solubilized from plaques. Plaque isolation protocols recover a small fraction of the plasma membrane connexin, with the bulk being solubilized by the detergent treatments and discarded. Thus, hemichannel preparations likely contain the bulk of the (detergent-soluble) junctional connexin as well as hemichannels that have not yet assembled into junctional channels. Conversely, the material in the isolated plaques is likely not represented in the hemichannel preparations, due to it being obtained on the basis of detergent *in*solubility. The basis for detergent insolubility of the small fraction of the plaques thus obtained is unknown.

Working with both hemichannels and junctional plaques potentially permits an additional level of stringency in identifying phospholipids interacting strongly with connexin protein. The two purification protocols likely have some specificity regarding extraction of lipids that is superimposed upon, and perhaps a determinant of, the different channel structural forms that are obtained. In this view, the lipids that remain associated with each connexin isoform through *both *isolation protocols would be among those most confidently and rigorously viewed as being tightly bound to the connexin (designated with ϕ in Tables 1 to 5). In addition, it may be that the plaque structure would not be maintained if the lipids most tightly associated with the channels were removed. For example, removal of specific tightly associated lipids from the light-harvesting complex of photosystem II eliminates the ability to form two-dimensional or three-dimensional crystals [59]. This line of reasoning, if correct, makes these identified phospholipids of particular interest, but does not exclude that other lipids present and not identified here are just as important.

It may also be possible that some of the observed differences in the phospholipids bound to Cx26 and Cx32 channels arise from the cell lines expressing each connexin having a different cohort of phospholipid species. There is no indication that bears on whether this occurs, but it is possible. Since these experiments deal only with tight interactions between lipid and connexin channels, if such differential phospholipid synthesis and/or expression occurs, it would influence the findings only for lipid species whose synthesis is turned on or off, not those whose levels are reasonably modulated (for specific lipid-protein interactions, only small amounts in the membrane are required), and that interact tightly with connexin protein. Given these considerations, these factors are unlikely to have a significant influence on the reported results.

### Phospholipid associations with connexin

Lipids can affect membrane protein structure and function in many ways. The bulk properties of the bilayer that do so include the order-disorder of acyl chains, membrane 'fluidity', lateral pressure and/or curvature stress. Influences increasingly proximal to a membrane protein include surface charge, interactions with 'annular' lipids, and 'non-annular' or 'cofactor' lipids that bind tightly within a protein or at protein-protein interfaces [1,54,60,61]. Previous work has led to some inferences about the effects of bulk bilayer properties on connexin channels [15,62], although these studies were limited by the difficulties associated with working with preparations of native membrane or with cells.

In the present study, one presumes that annular and non-annular lipids would be present in both preparations, and that other lipids may be as well. The intact hemichannels in detergent micelles are immobilized on a bead matrix and washed slowly and extensively with 240 equiv vol of high and low ionic strength buffers containing 80 mM OG detergent (approx. 3–5× CMC) and no lipid. Initially, there may be some non-specific lipid in these micelles. However, in lipid-OG mixed micelles, at 80 mM OG, the average number of lipid molecules per micelle is effectively zero for 250 μM lipid [63]. More important, lipid exchange between micelles is very rapid, particularly when the micelle concentration is high (that is, when the detergent concentration is well above the CMC [64,65]), as is the case here (OG CMC is approx. 16 to 23 mM). Additionally, all results excluded lipids identified in the negative controls, that is, for hemichannels, non-specific retention on immunobeads; for plaques, presence in material from HeLa cells that did not express connexin. Thus, lipid not specifically interacting with connexin would be rapidly removed during the extensive rinsing of the immobilized channels, and any lipid remaining in the micelles is there because it remains tightly associated with the connexin.

Isolated junctional plaques can be considered two-dimensional crystals in native lipid, similar to those used in structural analysis that are formed by purified proteins to which native lipids have been added [66], or that are isolated directly from native membranes [67]. Since plaque isolation protocols remove substantial lipid from native junctions [10], at least some of the identified lipid that remains is likely to be essential for the plaque structure.

Lipid removal is reflected in a decrease of the average lattice constant of the arrays of channels. In plasma membrane preparations, the lattice constant of junctional particles is approx. 90 to 95 Å [68,69]. Cx26 plaques isolated from the same HeLa cell line by the protocol used in this study have an average lattice constant of 76.2 ± 1.8 Å [23] (and the lattice constant ranges from 80 to 83 Å for junctional plaques isolated from rat liver, which are primarily Cx32 channels [69,70]). Assuming the lipid content of the plaques is reflected in the surface area *not *occupied by the channels, for a hexagonal lattice constant of 76 Å and junctional channel diameter within the bilayer of 63 Å [71], the decrease in lattice constant for the Cx26 plaques corresponds to loss of 52% to 60% of the lipids originally present. For a lipid headgroup area of 68 Å^2 ^[72], this corresponds to 27 lipid molecules remaining per Cx26 channel per leaflet.

Geometric calculation of the number of boundary lipids, a method validated by experimental data [4], yields 24 lipid molecules remaining per Cx26 channel per leaflet. Thus, it can be reasonably argued that the phospholipids identified here in the isolated plaques are predominantly annular and non-annular lipids, plus perhaps some additional lipids essential for plaque integrity. A caveat to this analysis is that some lipids that mediate interactions within highly crystalline two-dimensional structures may not fulfill the same roles *in vivo *[73].

For the reasons outlined above, in the present study, the lipids that are identified in the hemichannels and plaques are most likely to influence the channels at the local level, and not by 'bulk lipid' effects. The effects of lipids shown in the TSF activity studies, on the other hand, could occur by either mechanism.

#### Hemichannels

Cx32 hemichannels are associated with unique species of PC, which Cx26 hemichannels are not, and Cx26 hemichannels are associated with unique species of PS, which Cx32 hemichannels are not. The proportion of charged lipid species is much greater among the phospholipids specific for each connexin compared with those lipids in common. The proportion of ether (*e*+*p*) glycerol-alkyl chain linkages is greatest for Cx32 hemichannel specific phospholipids. Cx26 phospholipids have a bimodal distribution of desaturations, and for Cx32-specific phospholipids the shorter chain lengths tend to be more saturated.

The data suggest that specific PC species are involved in Cx32-specific structural and/or regulatory interactions but that PCs do not have Cx26-specific interactions. Similarly, the data suggest that specific forms of PS have Cx26-specific structural and/or regulatory interactions, as no PS species have Cx32-specific interactions.

#### Plaques

No species of PI or PS are shared between Cx26 and Cx32 plaques. The phospholipids unique to Cx32 plaques tend to have a greater proportion of charged species than those unique to Cx26 plaques. The phospholipids retained in all the plaque preparations have a broad distribution of saturations, but for Cx26 plaques the shorter chains tend to be more saturated. The proportion of Cx26-specific ether-linked phospholipids is higher for the Cx32-specific lipids.

These results suggest that plaques, in general, require phospholipids with a broad distribution of saturations and chain lengths, do not require PS or PI for structural integrity, and may require specific varieties of PC, PE, and PA.

The first compositional analyses of the lipids in gap junctions (using different protocols than this study) were reported for rodent liver [11,74], which contain Cx26 and Cx32. PC was identified as the major phospholipid, with significant PS and lesser amounts of PE and PI. Significant cholesterol was present. Other work reported that cholesterol is specifically enriched in junctional plaques [10]. Cholesterol supplementation of cultured cells was shown to increase gap junction formation [75], which subsequently was attributed to indirect effects [76]. Lens fiber junctions (composed of Cx46 and Cx50), were reported to contain significant sphingomyelin, cholesterol, large amounts of PC and PE and smaller amounts of PS [16]. Our data are consistent with these studies, and provide substantial additional detail about the specific phospholipid species involved. It was recently reported that maturation of gap junctions as they move to the inner cortical regions of the lens is associated with near complete removal of cholesterol from the junctional membranes [77]. The reason for this is unknown, and it may be specific to the lens, and/or to Cx46 and Cx50.

The literature on lipids associated with two-dimensional arrays of proteins in native membranes is largely limited to bacterial and thylakoid membranes. This literature established that the lipids tightly associated with such arrays are structurally and/or functionally important. For example, 'delipidation' of bacteriorhodopsin identified various species of PG, in particular, as being associated with the purple membrane structure [57]. Specific forms of PG are required for two-dimensional crystal formation of the protein [78]. Other work on bacteriorhodopsin, light harvesting complex II, and photosynthetic reaction centers show that PG is present in the three-dimensional crystal structures and is important for function [59,73,79,80]. Bacterial and mitochondrial membranes contain substantial PG, but it is absent from plasma membrane of eukaryotic cells, so one does not expect PG to play an analogous role in gap junction plaques.

#### Hemichannels and plaques

As described above, potentially the most stringent criterion for identifying tightly associated phospholipids is their presence in both hemichannel preparations and isolated plaques. In this category, the phospholipids unique to Cx26 are all anionic (PA and PI) and polyunsaturated. In contrast, phospholipids unique to Cx32 in both preparations are both anionic and zwitterionic (PE and PA), the former substantially more saturated and with longer acyl chain length than the latter. It is particularly striking that *all *of the phospholipids common to both connexin isoforms and structural forms are zwitterionic (PC and PE).

As species of PC and PE are found in common for both connexins, and in both the hemichannel and plaque preparations, one may infer that these phospholipids have strong interactions with both Cx26 and Cx32. As species of PA are uniquely found with each connexin in both hemichannel and plaque preparations, it can be inferred that different PAs interact strongly with Cx26 and Cx32. The differences that distinguish these lipids are that the acyl chains of the PA that interact specifically with Cx26 have fewer carbons and are less desaturated than those that interact specifically with Cx32. The fact that specific PEs are found in common suggests a particular relationship with 'non-bilayer' lipids.

### Properties of phospholipids associated with connexin

#### Membrane asymmetry

The plasma membrane of eukaryotic cells is asymmetric; the bulk neutral lipid and PC are in the outer leaflet, and anionic phospholipids plus PE are in the inner leaflet (refer to [81,82]). All the phospholipids found *uniquely *with each connexin isoform in both the hemichannel preparations and plaques (that is, potentially those most tightly and/or stringently associated with connexin) are from the inner leaflet, PA and PI for Cx26, and PA and PE for Cx32. The lipids found *in common *in both hemichannel preparations and isolated plaques were zwitterionic, PC and PE. The former suggests that the most important phospholipids that are specific for Cx26 or Cx32 are in the inner leaflet, and the latter suggests that there is a shared strong affinity for zwitterionic phospholipids in both leaflets. This kind of specificity of protein-lipid interactions for the phospholipids in the cytoplasmic leaflet is also seen for the KcsA channel, for which the phospholipid interactions in the extracellular leaflet are non-specific and in the cytoplasmic leaflet are specific (for PA and PG) [83].

#### Ether versus ester glycerol-alkyl chain linkage

The data show that Cx32 hemichannels are uniquely associated with substantially more species of ether-linked (*e*+*p*) phospholipids than Cx26 hemichannels (Figure 1b), while the reverse is true for plaques (Figure 2b). Ether-linked phospholipids have a variety of biological functions; most is known about the plasmalogens ('*p*' in the tables), which contain a vinyl ether bond at the *sn*-1 position [84]. Plasmalogens are membrane-delimited antioxidants and targets for oxidative damage, in addition to being regulators of membrane dynamics and reservoirs for polyunsaturated fatty acids [85,86]. Little is known about the specific membrane biophysics of plasmalogens, with the exception of ethanolamine (PE) plasmalogen, which, in contrast to other plasmalogens, promotes formation of hexagonal lipid phases (and thus, increased curvature stress; see below), to an even greater extent than ester-linked PE [87]. This is likely due to additional attractive forces between the headgroups arising from the decreased hydrophilicity from the lack of a carbonyl oxygen in the *sn*-1 chain. In this context it is interesting to note that the PE ether-linked lipids in the plaques were unique for Cx26, none being associated with Cx32 plaques (Table 3b). This could indicate that Cx26 plaques, as opposed to Cx32 plaques, have a particular requirement for the influence of non-bilayer lipids.

### Implications of activity studies

#### Surface charge

The activity data revealed a strong dependence on the magnitude of negative charge of the liposome membrane, independent of the specific headgroup contributing the charge. This is an important new finding regarding connexin channel function. It is particularly interesting in light of the finding that the phospholipids uniquely associated with either Cx26 or Cx32 hemichannels are mostly anionic (Table 6, Figures 3b and 3c). In addition, there is a correlation between the greater effect of anionic charge on Cx26 channel activity than Cx32 activity (Figure 6a, bars 3 and 4) and the greater number of anionic phospholipid species identified associated with Cx26 in hemichannels and plaques than with the corresponding Cx32 channels (Table 6 versus 7, Figures 3b and 3c and 4b and 4c).

The density of net positively charged residues in the cytoplasmic domains is greater for Cx26 than for Cx32, both in terms of average charge per length and concentration of positive charge in short segments. The recently crystal structure of Cx26 identifies the two transmembrane helices forming the outer wall of the pore (that is, exposed to membrane lipid) as being TM3 and TM4 [88]. Inspection of the relevant sequences suggests more net positive charges at the cytoplasmic membrane interface than at the extracellular one, but no particular differences between Cx26 and Cx32 in this regard.

Similar to our findings with connexin channels, pure PC membranes do not support significant acetylcholine receptor (AChR) activity, but the presence of anionic phospholipids restores function [89-93]. More recent studies stress the importance of acidic phospholipids as well as cholesterol (see below) in AChR function [94,95].

Surface charge can affect membrane protein function by a variety of mechanisms, in addition to affecting the voltage sensed by the protein. For example, activity of the reconstituted sugar transporter of the human erythrocyte increases with magnitude of surface charge in a manner suggesting it protects the catalytic domain against changes in membrane fluidity [96]. In this case, surface charge modulates the extent to which the state of the bilayer hydrocarbon chains affects transport activity.

The phospholipid compositions tested for their effects on Cx26 and Cx32 hemichannels also vary in their degree of curvature stress. In particular, PE and, to a lesser extent, PA contribute substantial negative curvature stress, while PS tends to oppose it [97]. Curvature stress of a membrane is approximately a linear function of the contribution of each component [98]. However, the distribution of phospholipids in each bilayer leaflet of liposomes is generally presumed to be identical, in which case the lipids themselves would not impose curvature stress. The situation is further complicated by the curvature of the small liposomes involved, and the consequences of that on the distribution of lipids. Thus, plotting the observed TSF activity data as a function of the calculated negative curvature potential of the bulk lipid has little meaning (doing so suggests a weak relationship where less calculated 'curvature' stress correlates with greater activity; not shown); the influence of curvature stress on connexin channel function induced by different phospholipids must be explored by other means.

#### Cholesterol

The data show that connexin channel activity in PC:cholesterol membranes is maximal at approx. 25 mol% cholesterol. Cholesterol interdigitates between bilayer lipids, modifying various physical properties of membranes [99], any or all of which could influence channel activity. Cholesterol may also have direct effects on membrane proteins. The simplest interpretations of the biphasic effect observed with increasing concentration (Figure 7) are either that cholesterol has two effects, one positive and one negative, with increasing concentration, or that a single parameter of its membrane effects has a biphasic modulatory influence.

Prominent among the effects of cholesterol on membrane properties are increased ordering of acyl chains [100], thickening of the bilayer [101] and increased dipole potential [102]. Increased acyl chain order would be expected to have a monotonic inhibitory effect on channel activity. For example, if a conformational change of a protein involved a change in its volume within the bilayer, increased order induced by cholesterol would inhibit that transition [103]. If an effect on acyl chain order contributes to the inhibitory phase of a biphasic effect, the activating phase would need to be caused by another factor, such as direct interaction with cholesterol. This explanation has been proposed for the biphasic effects of cholesterol on other membrane proteins, in which activation is caused by direct interaction with cholesterol and inhibition is caused by increased acyl chain order [104-106]. Alternatively, bilayer thickening could pass through an optimum for channel function as the hydrophobic thickness matches that of the channel protein in one or more of its conformational states. A role for hydrophobic match/mismatch between bilayer and membrane protein in protein function is gaining attention [4,107] (see below).

As the membrane concentration of cholesterol increases, so does the extent of interaction with phospholipid until virtually all the phospholipid capable of forming a condensed complex with cholesterol is interacting directly with it [108-111]. Above this concentration, the effects on membrane thickness level off [101,112,113], and pools of 'free' cholesterol form, that is, unassociated with phospholipid. The concentration at which this occurs varies with phospholipid species present, acyl chain length and degree of saturation, but often occurs at or near the concentration at which the maximal connexin channel activity was observed (approx. 25 mol% in PC). Therefore, in addition to its effects on bilayer structure and dynamics mentioned above, the decrease in channel activity at higher cholesterol concentrations could arise from increased direct protein-lipid interactions, with 'free' cholesterol having an inhibitory effect. The formation of such domains has been suggested to underlie a biphasic effect of cholesterol on membrane dipole potential that peaks approx. 25 mol% in dipalmitoyl-PC [102].

A peak in connexin channel activity at maximal cholesterol-induced membrane thickness could have important biological implications. Membrane thickness steadily increases in the secretory pathway from endoplasmic reticulum to Golgi to plasma membrane, due in part to increasing levels of cholesterol (less than 5 mol% to approx. 30 mol% [114]). Thus, cholesterol-mediated membrane thickening could play a role in connexin channel maturation to a functional state as it reaches the plasma membrane. As previously noted, unapposed connexin channels can exist in plasma membrane microdomains [49-51] that are typically thicker than surrounding membrane because of sphingomyelin and cholesterol enrichment [99]. Several lines of evidence also suggest substantial enrichment in the cholesterol/phospholipid ratio in gap junction plaques [9-16]. In addition, a connexin channel shifting between different microdomains, or between microdomains and plaques, could experience different types of hydrophobic mismatch with its surrounding bilayer, which could affect its function (see below).

### Lipid environment and connexins

#### Bilayer pressure profile

Though the weak positive effect of PE on channel activity is consistent with the effects of its small surface charge (Figure 6b), it is possible that PE also affects the channels via its ability to introduce changes in the curvature stress and lateral pressure profile of the bilayer. Forms of PE were found uniquely, and in common, for each connexin and for hemichannel *and *plaque preparations.

PE is a classic 'non-bilayer' lipid. Its small headgroup relative to the cross section of its acyl chains results in a conical shape that introduces negative curvature strain into membranes, that is, increased lateral pressure in the acyl chain region and decreased lateral pressure between the headgroups. As noted, PA has a similar but weaker effect, PS tends to oppose it [97], and curvature stress of a membrane is approximately a linear function of the contribution of each component [98]. The presence of such forces, primarily due to PE, could affect channel function, depending on the types of intramembrane volume changes that occur during gating [115-119]. Also, there may be a highly local relation between spontaneous curvature of the bilayer and the mean curvature of the lipid/water interface adjacent to the protein, as occurs for rhodopsin [120], a variation of the forces that come into play for 'hydrophobic mismatch', mentioned above and discussed below.

In this context it is interesting to note that the tendency of PE-containing membranes to form hexagonal phases, and the consequent increased membrane curvature stress, is enhanced by virtually all of the lipophiles that inhibit connexin channels (see below). These include certain alkanols [121], highly unsaturated fatty acids [122] (including oleamide, a connexin channel inhibitor), and halothane [123]. These agents may act synergistically with PE and other bilayer factors to affect connexin channel function via effects on the lateral membrane pressure profile. A possible exception is cholesterol, which can have the same effect on PE-containing membranes [124-126], but which enhanced channel activity in PC membranes in our study.

The ability of PE to promote negative curvature correlates with acyl chain length, and to a lesser degree with unsaturation [127]. The median chain lengths of the PE species specific to Cx26 plaque preparations are substantially greater than those in any of the other hemichannel or plaque preparations (approx. 40 versus approx. 36), suggesting a particular requirement for, or sensitivity to, negative curvature stress for Cx26.

In fact, the recent high-resolution crystal structure of a Cx26 channel indicates that each hemichannel is a toroid, approx. 30 Å wider at the cytoplasmic lipid boundary than at the extracellular lipid boundary [88]. This form would impose positive curvature stress to the bilayer, unless PE or other non-bilayer lipids were closely associated with the channel in the cytoplasmic leaflet, or lysolipids in the extracellular leaflet.

#### Hydrophobic mismatch and bilayer deformation energy

The PE-dependent effects on lateral bilayer stress described above are a special case of a more global aspect of membrane-protein energetics. Lateral bilayer stress can be induced and influenced by a variety of factors. Any reagent that partitions into a membrane and does not have a cylindrical shape or does not penetrate completely through the bilayer can affect the lateral stress via effects on intrinsic membrane curvature (the same parameter affected by PE). Of particular interest for connexin channels is the possibility of hydrophobic mismatch between the connexin channel and bilayer thickness, here suggested by the biphasic effect of cholesterol.

Hydrophobic mismatch interacts with the intrinsic membrane curvature to generate an energy that impinges on the channel, the 'bilayer deformation energy'. Consequently, membrane proteins can have optimal activity at specific average acyl chain lengths. Modeling shows that the effects arising from hydrophobic mismatch alone can account for biphasic relations between bilayer thickness and membrane protein activity [107,128].

The precise delimitations of the transmembrane helices of connexins remain uncertain; unlike many membrane proteins, the connexin amino acid sequence does not have a conspicuous clustering of aromatic residues at the boundaries between exposure to lipid headgroups and acyl tail [129,130]. However, the recent crystal structure of Cx26 suggests membrane boundaries of the transmembrane helices and a membrane thickness of approx. 38 Å [88]. The two external helices (TM3 and TM4) form the channel boundary with membrane lipid, and are exposed to lipid for almost all of their lengths that are within the membrane. These helices are tilted relative to each other and to the plane of the membrane. The crystal structure shows intraprotomer contacts between these two helices.

In this situation, change of the hydrophobic thickness of the surrounding bilayer will alter the tilt of the helices relative to each other, and relative to the channel axis. This structural change may be a dynamic and specific mechanism by which changes to the hydrophobic thickness of the surrounding bilayer can be sensed and transduced to modulate channel function. One could imagine, for example, such functional channel 'maturation' arising after increases (by cholesterol, see above, or other means) in hydrophobic thickness, for example, in the secretory pathway from endoplasmic reticulum to Golgi to plasma membrane.

#### Lipophilic modifiers of connexin activity

A predominance of the known modulators of gap junction communication are lipophilic and/or show some specificity with regard to membrane lipids [17,18]. Among the lipophiles that inhibit connexin channels are the aliphatic alcohols heptanol and octanol [131,132], volatile anesthetics such as halothane [133], arachidonic acid and other non-saturated fatty acids and their amides, for example, oleamide (*cis*-9-octadecenamide) [134,135], and the triterpinoid saponins, glycerrhetinic acid and carbenoxolone [136]. These reagents are not selective for connexin channels [17,18], and likely act by partitioning into plasma membrane and affecting membrane physical properties. The present study suggests that the reported differences and apparently contradictory reports of the effects of specific lipophiles on different connexin isoforms [18] may arise, in part, from the distinct sets of lipids that are closely associated with different connexin isoforms.

Aliphatic alcohols produce a rapid and reversible inhibition of gap junctional conductance [131], with potency inversely related to chain length. The effects of heptanol and octanol are specifically correlated with a decrease in the fluidity of the cholesterol-rich domains (that is, plaques) in which junctional channels reside, in spite of an increase in bulk membrane fluidity elsewhere [132]. *Cis*-unsaturated fatty acids, including oleamide, affect connexin channels in a manner positively correlated with the degree of unsaturation and, like aliphatic alcohols, negatively correlated with chain length [137,138]. The ability of fatty acids to affect the function of membrane proteins is thought to be due to their ability to increase disorder of the lipid acyl chains of the membrane [139].

It is unclear whether *cis*-unsaturated fatty acids have different effects on cholesterol-rich and cholesterol-poor membranes, as do the aliphatic alcohols. In both cases, the relevant membrane parameter, at least regarding bulk membrane properties, may be modification of the lateral pressure profile rather than acyl chain order/disorder *per se*. In addition, different fatty acids affect different connexins in distinct ways, perhaps reflecting different efficacies for acyl chain disruption of specific lipid species, consistent with our observed preference of different connexins to associate with different phospholipids.

The inhalation anesthetic halothane rapidly and reversibly inhibits junctional communication [131], but does so with junctional channels formed by heteromeric Cx40/Cx43 hemichannels at significantly lower doses than either of the corresponding homomeric Cx40 or Cx43 hemichannels, demonstrating that halothane sensitivity can be influenced by connexin isoform composition [133]. Halothane binds with high affinity to appropriately sized hydrophobic cavities in proteins to stabilize helix-helix configurations [140]. While this is a possible mechanism for its different effects on these homomeric and heteromeric channels, it could also arise from effects on membrane physical properties, if the lipid micro-environments around homomeric and heteromeric channels are different, as inferred from the present study.

## Conclusion

The data show that Cx26 and Cx32 hemichannels have a preference for tight association with unique anionic phospholipids, and that anionic phospholipids, independent of headgroup, have a positive effect on activity of both Cx26 and Cx32 channels. All the anionic phospholipids retained in both the hemichannel preparations and isolated plaques were specific for either Cx26 or Cx32. These were species of PA and PI for Cx26, and PA and PE for Cx32. The difference between the PAs specific for each isoform suggests that Cx26 prefers PAs with longer acyl chain lengths and more desaturations. For the hemichannels there were connexin-specific interactions for a variety of phospholipids; no species of PC interacted specifically with Cx26, and no species of PS interacted specifically with Cx32. From the analysis of headgroups, it appears that the likely phospholipid modulators of connexin channel structure-function that are connexin isoform-specific are in the cytoplasmic leaflet. A role for phospholipids that promote negative curvature (for example, PE) is inferred.

Cholesterol has a biphasic effect on Cx26 and Cx32 channel activity, but it was not possible to distinguish among its effects on membrane thickness (hydrophobic mismatch), lateral pressure profile, acyl chain order or membrane dipole for channel activity.

PC and PE species were found in common in both hemichannel preparations and isolated plaques of both connexins. The phospholipids found uniquely with each connexin tended to differ from this pattern, which could suggest that changes in the ratio of PE to PC could modulate the channels via changes in negative membrane curvature.

While only two connexins and a limited set of phospholipids have been studied here, the key 'proof-of-principle' finding is that connexins, even two with significant sequence homology, are likely to have distinct lipid 'affinities' as both isolated hemichannels and as junctional plaques. There are also lipids associated with both isoforms and channel structures. The differences and similarities suggest modes of lipid influence on respective structural forms. The fact that this study identified endogenous lipids interacting with two connexin isoforms adds biological credibility. However, in both the lipid analysis and functional study, only positive findings have meaning: other lipids may be involved in channel structure, and there may be effects of membrane composition on channel function not revealed by the TSF liposome assay.

Nevertheless, these experiments make clear that the activity and modulation of connexin channels should begin to be considered within the context of the lipids comprising the membrane, whether within junctional plaques or surrounding unpaired hemichannels. This will be true not only for a detailed molecular description of their activity but also for understanding the mechanisms and forces that affect channel activity. Such understanding will require, in addition to higher resolution structural information, determination of the specificity of binding of individual versus bulk lipids, the location of their binding sites, and the occupancies and cooperativity of any/all potential interactions with connexins.

## Abbreviations

AChR: acetylcholine receptor; amu: atomic mass units; CMC: critical micelle concentration; Cx26: connexin26; Cx32: connexin32; DFP: diisopropyl fluorophosphate; DOPE: dioleoylphosphatidylethanolamine; DOPE-NBD: DOPE labeled with nitrobenzoxadiazole; DOPE-rhodamine: DOPE labeled with lissamine rhodamine B; EDTA: ethylenediaminetetraacetic acid; EGTA: ethylene glycol tetraacetic acid; ESI-MS/MS: tandem electrospray-mass spectroscopy; HA: haemagglutinin epitope; HEPES: 4-(2-hydroxyethyl)-1-piperazineethanesulfonic acid; IgG: immunoglobulin G; OG: *n*-octyl βD glucopyranoside; PA: L-α-phosphatidic acid; PC: L-α-phosphatidylcholine; PE: L-α-phosphatidylethanolamine; PG: L-α-phosphatidylglycerol; PI: L-α-phosphatidylinositol; PS: L-α-phosphatidylserine; TSF: Transport Specific Fractionation

## Authors' contributions

DL conceived, designed and performed the experiments. DL and ALH analyzed the data, and wrote the manuscript.
